# Does CMR improve aetiological sub-phenotyping beyond echocardiography in patients with elevated LV filling pressure? A prospective registry study (PREFER-CMR)

**DOI:** 10.1136/bmjopen-2025-102836

**Published:** 2026-01-14

**Authors:** Aradhai Bana, Rui Li, Zia Mehmood, Craig Rogers, Ciaran Grafton-Clarke, Tiya Bali, David Hall, Mustapha Jamil, Liandra Ramachenderam, Uwais Dudhiya, Hilmar Spohr, Victoria Underwood, Rebekah Girling, Bahman Kasmai, Sunil Nair, Gareth Matthews, Pankaj Garg

**Affiliations:** 1 University of East Anglia, Norwich, UK; 2 Norfolk and Norwich University Hospitals NHS Foundation Trust, Norwich, UK

**Keywords:** Heart failure, Echocardiography, Magnetic resonance imaging

## Abstract

**Abstract:**

**Objectives:**

To evaluate the incremental diagnostic value and sub-phenotyping capability of Cardiovascular Magnetic Resonance (CMR) compared with Transthoracic Echocardiography (TTE) in patients with elevated left ventricular filling pressure (LVFP).

**Design:**

Prospective registry study. [Results from ClinicalTrials.gov ID NCT05114785]

**Setting:**

A single NHS hospital in the UK.

**Main outcome measures:**

The primary outcome was the rate of diagnostic discordance between TTE and CMR. Secondary outcomes included the characterisation of specific pathologies identified by CMR where TTE was normal, non-diagnostic or provided a non-specific diagnosis.

**Results:**

CMR demonstrated diagnostic discordance with TTE in 74% (n=194) of cases. In patients with a normal TTE (n=54), CMR identified heart failure with preserved ejection fraction (HFpEF) in 46% (n=25) and ischaemic heart disease (IHD) in 19% (n=10). For non-diagnostic TTE cases (n=15), CMR detected HFpEF in 53.3% (n=8) and IHD in 26.7% (n=4). Among those with non-specific left ventricular hypertrophy on TTE (n=47), CMR revealed HFpEF in 45% (n=21) and hypertrophic cardiomyopathy in 34% (n=16).

**Conclusions:**

CMR markedly improves diagnostic precision and sub-phenotyping in patients with elevated LVFP, identifying key conditions like HFpEF, IHD and specific cardiomyopathies that TTE frequently misses. These findings highlight CMR’s critical role as a complementary imaging tool for refining diagnoses and informing management strategies in cardiovascular conditions.

**STRENGTHS AND LIMITATIONS OF THIS STUDY**

- Prospective design with data from a real-world clinical registry, which reflects current clinical practice.
- The ‘all-comers’ approach for the registry enhances the generalisability of findings to patients who are referred for multi-modality imaging due to diagnostic uncertainty.
- A key limitation is that the cohort was derived from a single centre, which may introduce referral bias and limit applicability to other settings.
- Left ventricular filling pressure was estimated using a non-invasive Cardiovascular Magnetic Resonance (CMR)-derived equation without validation against the gold standard of invasive catheterisation.
- The study population included only patients referred for both Transthoracic Echocardiography and CMR, potentially skewing the cohort towards more diagnostically challenging cases.

## Introduction

 Cardiovascular diseases remain a leading cause of morbidity and mortality worldwide, with heart failure (HF) representing a significant burden due to its heterogeneous pathophysiology and diagnostic challenges.^1^ Accurate phenotyping of cardiac conditions, particularly those involving elevated left ventricular filling pressure (LVFP), is critical for tailoring therapeutic interventions and improving patient outcomes. Transthoracic echocardiography (TTE) has long been the cornerstone of non-invasive cardiac assessment, offering real-time insights into structural and functional abnormalities.^2^ However, its limitations in detecting myocardial pathology, such as fibrosis, inflammation, scar or early diastolic dysfunction, often result in nonspecific or inconclusive diagnosis.^3^

Cardiovascular magnetic resonance (CMR) has emerged as a powerful tool, providing myocardial tissue characterisation, precise volumetric quantification and the ability to assess myocardial scar and extracellular matrix expansion through techniques like late gadolinium enhancement (LGE) and T1 mapping.^4^,^6^ Despite its potential, the comparative diagnostic yield of CMR over TTE^7^ in patients with raised LVFP remains underexplored, particularly in prospective, real-world cohorts. Previous studies have suggested that CMR-derived metrics, such as pulmonary capillary wedge pressure (PCWP) estimated from left atrial volume (LAV) and left ventricular mass (LVM), offer haemodynamic insights that correlate with invasive measurements.^8^,^13^ Yet, the extent to which CMR can resolve diagnostic discrepancies or uncover occult pathologies missed by TTE has not been systematically evaluated. This gap is particularly pertinent in high-risk populations where precise diagnosis can alter therapeutic trajectories, such as in heart failure with preserved ejection fraction (HFpEF), ischaemic heart disease (IHD) or infiltrative cardiomyopathies.

For this study, we hypothesised that CMR would provide greater diagnostic precision and sub-phenotyping capability compared with TTE in patients with elevated LVFP, identifying specific pathologies where TTE yields normal or non-diagnostic findings.

The main objective of this study was to evaluate the incremental diagnostic value of CMR over TTE in a prospective cohort from the PREFER-CMR registry, focusing on patients with CMR-derived LVFP exceeding 14 mm Hg.

## Methods

### Study population

This study used data from the prospective PREFER-CMR registry (ClinicalTrials.gov: NCT05114785) to identify eligible participants. It was designed as an all-comers study, including individuals who underwent TTE followed by CMR imaging between February 2022 and July 2024.

### Inclusion criteria

Participants were required to be at least 18 years of age and to have undergone TTE as part of their diagnostic assessment, followed by a clinically indicated CMR. For the purpose of this focused investigation, only individuals with CMR-derived LVFP exceeding 14 mm Hg were included.

### Exclusion criteria

Exclusion criteria encompassed individuals with contraindications to CMR, including those with non-compatible implanted defibrillators, severe claustrophobia or advanced renal dysfunction (eGFR <30 mL/min/1.73 m²).

### Patient involvement

Patients or the public were not involved in the design, or conduct, or reporting, or dissemination plans of our research.

### Transthoracic echocardiography

Standard TTE was performed in accordance with the guidelines set by the British Society of Echocardiography. The evaluation focused on key parameters essential for assessing LVFP, including the ratio of early diastolic mitral inflow velocity to early diastolic mitral annular velocity (E/e’), the early diastolic mitral annular velocity (e’), tricuspid regurgitation (TR) peak velocity, septal thickness and left atrial volume. These echocardiographic indices were systematically measured to provide a comprehensive assessment of diastolic function and ventricular compliance. All echocardiographic measurements were conducted by trained sonographers and reviewed by experienced cardiologists to ensure accuracy and adherence to standardised protocols.

As part of this standardised protocol, echocardiographic image quality was formally graded by the reporting sonographer. In this framework, a ‘non-diagnostic’ study is specifically defined as a scan that is deemed by the sonographer to have ‘no diagnostic utility’. This is the most severe classification and is distinct from a study of ‘limited’ quality, which may provide some, but not all, of the required diagnostic information.

### CMR protocol and analysis

All CMR studies were performed using a 1.5 Tesla Magnetom Sola system (Siemens Healthineers, Erlangen, Germany), equipped with BioMatrix Body 18 coil technology to enhance imaging precision and patient adaptability. The standardised CMR protocol encompassed baseline localisation surveys followed by cine imaging sequences to assess cardiac structure and function. These cine sequences included vertical long-axis, horizontal long-axis and short-axis contiguous left ventricular volume stack acquisition. Cine images were obtained during end-expiratory breath-hold using a balanced steady-state free precession (bSSFP) single-slice breath-hold sequence. A total of 30-phase cine images were acquired with a contiguous slice thickness of 8 mm for the short-axis stack. Imaging parameters included an echo time (TE) of 1.13 ms, a repetition time (TR) of 2.71 ms, a flip angle of 80°, a field of view (FOV) of 360×289 mm², and a GRAPPA acceleration factor of 2. Myocardial T1 mapping was performed using a Modified Look-Locker Inversion Recovery sequence to quantify native myocardial T1 times. Extracellular volume (ECV%) was calculated from precontrast and postcontrast T1 mapping values, incorporating the patient’s haematocrit to estimate myocardial extracellular matrix expansion. Standard clinical LGE imaging was performed to assess focal myocardial scar or fibrosis, aiding in the final diagnosis and differentiation of cardiomyopathic phenotypes.

All imaging procedures were conducted by trained radiographers and analysed by experienced cardiologists and technicians with greater than 3 years of CMR experience in accordance with established CMR guidelines to maintain consistency and diagnostic accuracy. Furthermore, CMR data post-processing and interpretation was conducted independently and blinded to echocardiographic findings and other clinical parameters to eliminate potential bias and maintain the integrity of the study results.

CMR image analysis was performed using CVI 42 software (V.5.17.1, Circle Cardiovascular Imaging, Calgary, Canada). Left ventricular end-diastolic volume (mL), left ventricular end-systolic volume (mL), left ventricular stroke volume (mL), left ventricular ejection fraction (%), LVM in diastole (g), and LAV at end-systole (mL) were quantified using semi-automated contouring methods on short-axis cine images. Manual adjustments were performed as necessary to ensure precision. Left ventricular volumes and ejection fraction were derived using Simpson’s method of discs. LVM was calculated by subtracting the endocardial volume from the epicardial volume and multiplying by myocardial density.

#### Estimation of left ventricular filling pressure using sex-specific CMR-derived equations

To estimate LVFP, we used sex-specific equations derived from CMR metrics, as described previously. This equation incorporates LAV and LVM to calculate the PCWP, serving as a surrogate for LVFP.^9^ LAV was measured using the biplane area-length method from the 2-chamber and 4-chamber cine images at end-systole. LVM was determined through short-axis segmentation in end-diastole using established techniques. Papillary muscles were included in blood volume. These parameters were integrated into sex-specific equations to derive estimates of LVFP, providing a robust method for evaluating diastolic function. The sex-specific equation is as follows:

CMR PCWP=5.7591 + (0.07505×LAV) + (0.05289×LVM) – (1.9927×sex) [female=0; male=1]

Where:

- PCWP is the pulmonary capillary wedge pressure in mm Hg.
- LAV is the left atrial volume in mL.
- LVM is the left ventricular mass in g.

All CMR image analyses were conducted using dedicated research software (MASS version 2021-Exp, Leiden University Medical Centre, Leiden, the Netherlands). CMR contour tracings, including volume/function assessments and LGE segmentation, were performed by R.J.G at core-lab (Leiden).

### Statistical analysis

Statistical analyses were performed using MedCalc for Windows, V.23.1.7 (MedCalc Software, Ostend, Belgium). The large dataset was treated as parametric, assuming normality. Continuous variables were summarised as mean±SD, and categorical variables as freq (%) for parametric data. Normality was assumed due to large N, enabling parametric tests. To compare baseline demographics, TTE parameters and CMR findings between groups with similar versus non-similar diagnosis, we used t-tests for continuous variables and χ² tests for categorical variables. These tests were chosen due to large N, ensuring power and minimising errors. Diagnosis concordance between TTE and CMR was quantified as % of similar versus non-similar Diagnosis. Subgroup analyses assessed CMR Dx yield in TTE categories (normal, non-diagnostic, non-specific left ventricular hypertrophy (LVH), non-specific cardiomyopathy (CMP)). Prevalence of CMR-identified pathologies was reported as % with 95% CI. Significance was set at p<0.05 for all analyses, following standard clinical research thresholds.

## Results

### Study population

The study population comprised 261 individuals, with 194 (74%) having a non-similar diagnosis between modalities and 67 (26%) having a similar diagnosis (table 1). Males constituted 62% of the non-similar diagnosis group and 54% of the similar diagnosis group (p=0.24). The mean age was 57.7±16.2 years in the non-similar group and 61.5±13.8 years in the similar group (p=0.09). Body surface area was comparable between groups at 2.0±0.3 m² and 2.0±0.4 m², respectively (p=0.97). Estimated glomerular filtration rate showed no significant difference, with values of 77.4±15.7 mL/min/1.73 m² and 78.7±16.7 mL/min/1.73 m² (p=0.56). Creatinine levels were 86.6±40.1 µmol/L and 84.8±57.7 µmol/L (p=0.78), while haemoglobin levels were 141.5±15.1 g/L and 138.8±15.0 g/L (p=0.22). N-terminal pro B-type natriuretic peptide levels were 953.3±2473.9 pg/mL in the non-similar group and 1748.5±3922.0 pg/mL in the similar group (p=0.23). Hypertension was present in 42% and 43% of individuals, respectively (p=0.83), while diabetes mellitus was observed in 12% and 19% (p=0.16). Current smoking was reported in 10% of the non-similar group and 3% of the similar group (p=0.08). Cerebrovascular accident prevalence was 4% and 3% (p=0.81), and atrial fibrillation was more frequent in the similar diagnosis group at 24% compared with 13% in the non-similar group (p=0.04). Myocardial infarction occurred in 16% and 21% (p=0.42), while ventricular tachycardia was noted in 2% and 1% (p=0.98). Mean heart rates were 65.1±13.0 beats per minute and 64.8±11.6 beats per minute (p=0.88).

**Table 1:** Study demographics stratified as per similarity of final diagnosis by transthoracic echocardiography or cardiovascular magnetic resonance

	Not similar diagnosis between modalities n=194	Similar diagnosis between modalities n=67	P value
Sex (male)	120 (62%)	36 (54%)	0.24
Age (years)	57.7±16.2	61.5±13.8	0.09
Body Surface Area (m²)	2.0±0.3	2.0±0.4	0.97
Estimated Glomerular Filtration Rate (mL/min/1.73 m²)	77.4±15.7	78.7±16.7	0.56
Creatinine (µmol/L)	86.6±40.1	84.8±57.7	0.78
Haemoglobin (g/L)	141.5±15.1	138.8±15.0	0.22
N-terminal pro B-type Natriuretic Peptide (pg/mL)	953.3±2473.9	1748.5±3922.0	0.23
Hypertension (yes)	81 (42%)	29 (43%)	0.83
Diabetes Mellitus (yes)	24 (12%)	13 (19%)	0.16
Current smoker (yes)	19 (10%)	2 (3%)	0.08
Cerebrovascular accident (yes)	7 (4%)	2 (3%)	0.81
Atrial fibrillation (yes)	26 (13%)	16 (24%)	0.04
Myocardial infarction (yes)	32 (16%)	14 (21%)	0.42
Ventricular Tachycardia (yes)	3 (2%)	1 (1%)	0.98
Heart rate (beats per minute)	65.1±13.0	64.8±11.6	0.88

### Echocardiographic findings

Echocardiographic findings are presented in table 2. The LAV index was significantly higher in the similar diagnosis group at 42.2±21.1 mL/m² compared with 33.3±12.3 mL/m² in the non-similar group (p=0.0004). Early diastolic mitral inflow velocity was 0.9±2.6 cm/s and 2.0±9.4 cm/s (p=0.18), while late diastolic mitral inflow velocity was 1.6±9.0 cm/s and 3.3±17.6 cm/s (p=0.40). The early-to-late mitral inflow velocity ratio was 1.1±0.5 in the non-similar group and 1.0±0.4 in the similar group (p=0.35). The mitral inflow E velocity to mitral annular early diastolic velocity ratio was significantly higher in the similar diagnosis group at 10.5±5.3 compared with 8.7±3.4 in the non-similar group (p=0.004). Longitudinal early diastolic strain rate was 10.3±3.9 1/s and 9.2±3.9 1/s (p=0.09), while longitudinal systolic strain rate was 8.0±2.9 1/s and 7.2±2.8 1/s (p=0.05). TR velocity was comparable between groups at 2.4±0.5 m/s and 2.5±0.6 m/s (p=0.34). Interventricular septal thickness was also similar at 11.9±3.0 mm and 12.1±2.7 mm (p=0.69).

**Table 2:** Echocardiography findings in the study

	Not similar diagnosis between modalities	Similar diagnosis between modalities	P value
Left Atrial Volume Index (mL/m²)	33.3±12.3	42.2±21.1	0.0004
Early Diastolic Mitral Inflow Velocity (cm/s)	0.9±2.6	2.0±9.4	0.18
Late Diastolic Mitral Inflow Velocity (cm/s)	1.6±9.0	3.3±17.6	0.40
Early-to-Late Mitral Inflow Velocity Ratio (unitless)	1.1±0.5	1.0±0.4	0.35
Mitral inflow E velocity to mitral annular early diastolic velocity ratio (E/e′)	8.7±3.4	10.5±5.3	0.004
Longitudinal Early Diastolic Strain Rate (1/s)	10.3±3.9	9.2±3.9	0.09
Longitudinal Systolic Strain Rate (1/s)	8.0±2.9	7.2±2.8	0.05
Tricuspid Regurgitation Velocity (m/s)	2.4±0.5	2.5±0.6	0.34
Interventricular Septal Thickness (mm)	11.9±3.0	12.1±2.7	0.69

### CMR findings

CMR findings are presented in table 3. Left ventricular end-diastolic volume was 155.1±33.7 mL and 155.9±38.2 mL (p=0.87), while left ventricular end-systolic volume was 59.8±18.9 mL and 61.0±22.8 mL (p=0.69). Left ventricular stroke volume was comparable between groups at 95.0±20.6 mL and 95.2±24.3 mL (p=0.95), and left ventricular ejection fraction was 62.0±7.6% and 61.2±8.3% (p=0.51). LVM index was 144.5±41.9 g/m² in the non-similar group and 152.6±53.5 g/m² in the similar group (p=0.21). Right ventricular end-diastolic volume was 168.3±41.4 mL and 164.9±48.5 mL (p=0.58), while right ventricular end-systolic volume was 74.4±24.3 mL and 71.9±31.2 mL (p=0.50). Right ventricular stroke volume was 93.9±22.2 mL and 92.9±24.4 mL (p=0.74), with right ventricular ejection fraction at 56.3±6.7% and 57.4±9.0% (p=0.29). Myocardial native T1 relaxation time was 1014.2±66.4 ms in the non-similar group and 1026.0±54.0 ms in the similar group (p=0.19). EVF was 25.4±5.5% and 26.1±4.4% (p=0.29). LVFP was significantly higher in the similar diagnosis group at 20.5±4.7 mm Hg compared with 18.8±3.2 mm Hg in the non-similar group (p=0.001).

**Table 3:** Cardiovascular magnetic resonance findings for the whole cohort

	Not similar diagnosis between modalities	Similar diagnosis between modalities	P value
Left Ventricular Global Longitudinal Strain (%)	−0.2±0.05	−0.4±2.0	0.10
Left Ventricular End-Diastolic Volume (mL)	155.1±33.7	155.9±38.2	0.87
Left Ventricular End-Systolic Volume (mL)	59.8±18.9	61.0±22.8	0.69
Left Ventricular Stroke Volume (mL)	95.0±20.6	95.2±24.3	0.95
Left Ventricular Ejection Fraction (%)	62.0±7.6	61.2±8.3	0.51
Left Ventricular Mass Index (g/m²)	144.5±41.9	152.6±53.5	0.21
Right Ventricular End-Diastolic Volume (mL)	168.3±41.4	164.9±48.5	0.58
Right Ventricular End-Systolic Volume (mL)	74.4±24.3	71.9±31.2	0.50
Right Ventricular Stroke Volume (mL)	93.9±22.2	92.9±24.4	0.74
Right Ventricular Ejection Fraction (%)	56.3±6.7	57.4±9.0	0.29
Myocardial Native T1 Relaxation Time (ms)	1014.2±66.4	1026.0±54.0	0.19
Extracellular Volume Fraction (%)	25.4±5.5	26.1±4.4	0.29
Left Ventricular Filling Pressure (mm Hg)	18.8±3.2	20.5±4.7	0.001

### Echocardiographic diagnosis in patients with high CMR LVFP

**Figure 1:**
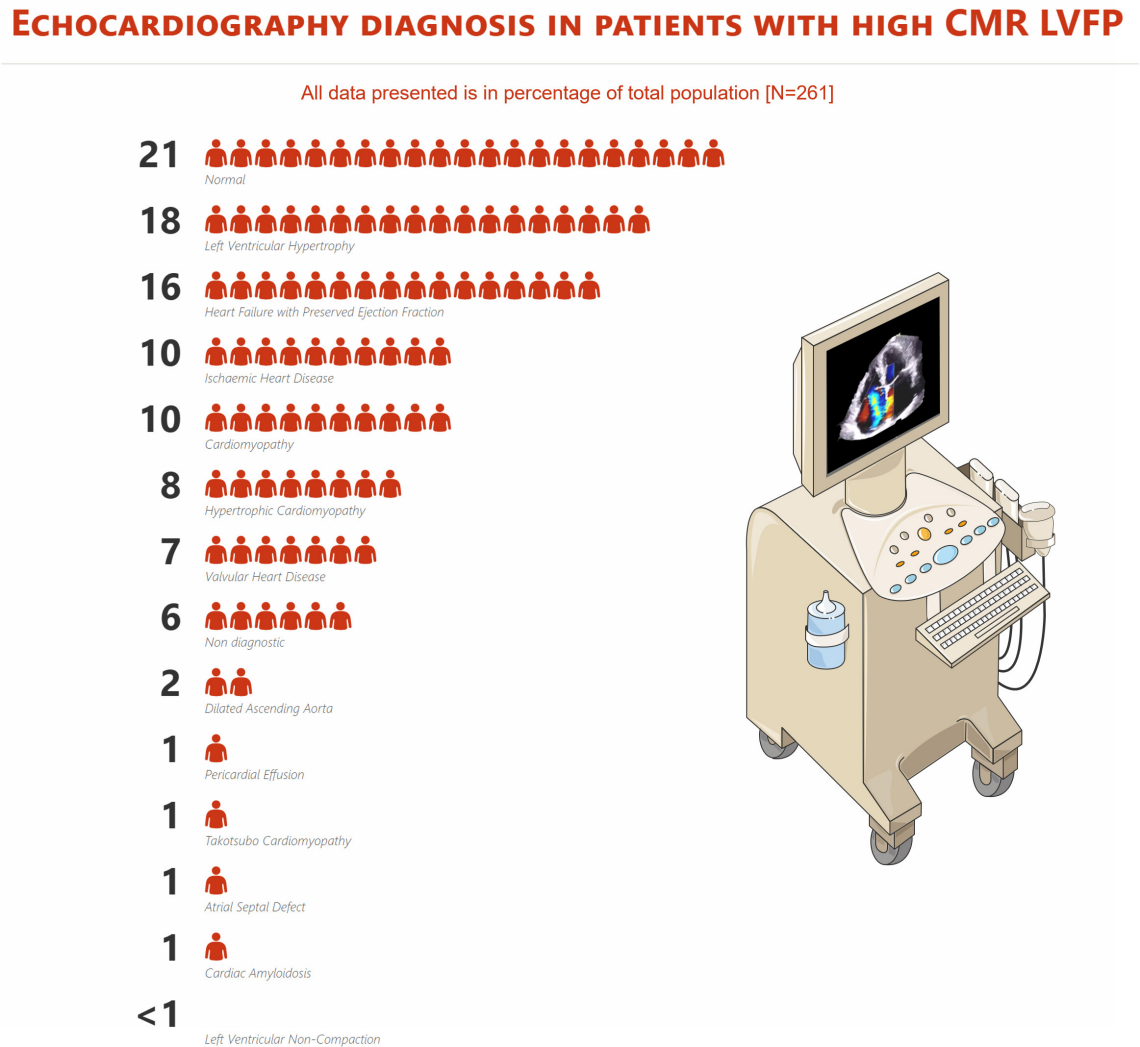
Echocardiographic diagnoses in patients with high CMR LVFP. Normal findings, LVH and HFpEF were most common, followed by IHD, cardiomyopathy and valvular disease. Less frequent diagnoses included structural and infiltrative conditions. CMR, cardiovascular magnetic resonance; LVFP, left ventricular filling pressure.

Among patients with high LVFPs on CMR, echocardiographic evaluation revealed normal findings in 21% (n=54) of cases (figure 1). LVH was observed in 18% (n=47) of patients, while HFpEF was identified in 16% (n=41). IHD and cardiomyopathy were each diagnosed in 10% (n=26 and n=25, respectively), followed by hypertrophic cardiomyopathy in 8% (n=20) and valvular heart disease in 7% (n=18). In 6% (n=15) of cases, echocardiography yielded non-diagnostic results. Less frequently, a dilated ascending aorta was reported in 2% (n=4), while atrial septal defect (n=2), cardiac amyloidosis (n=2), pericardial effusion (n=3) and Takotsubo cardiomyopathy (n=3) were each observed in 1% of cases. Left ventricular non-compaction was found in <1% (n=1) of patients.

### Normal echocardiography study in patients with raised LVFP by CMR

**Figure 2:**
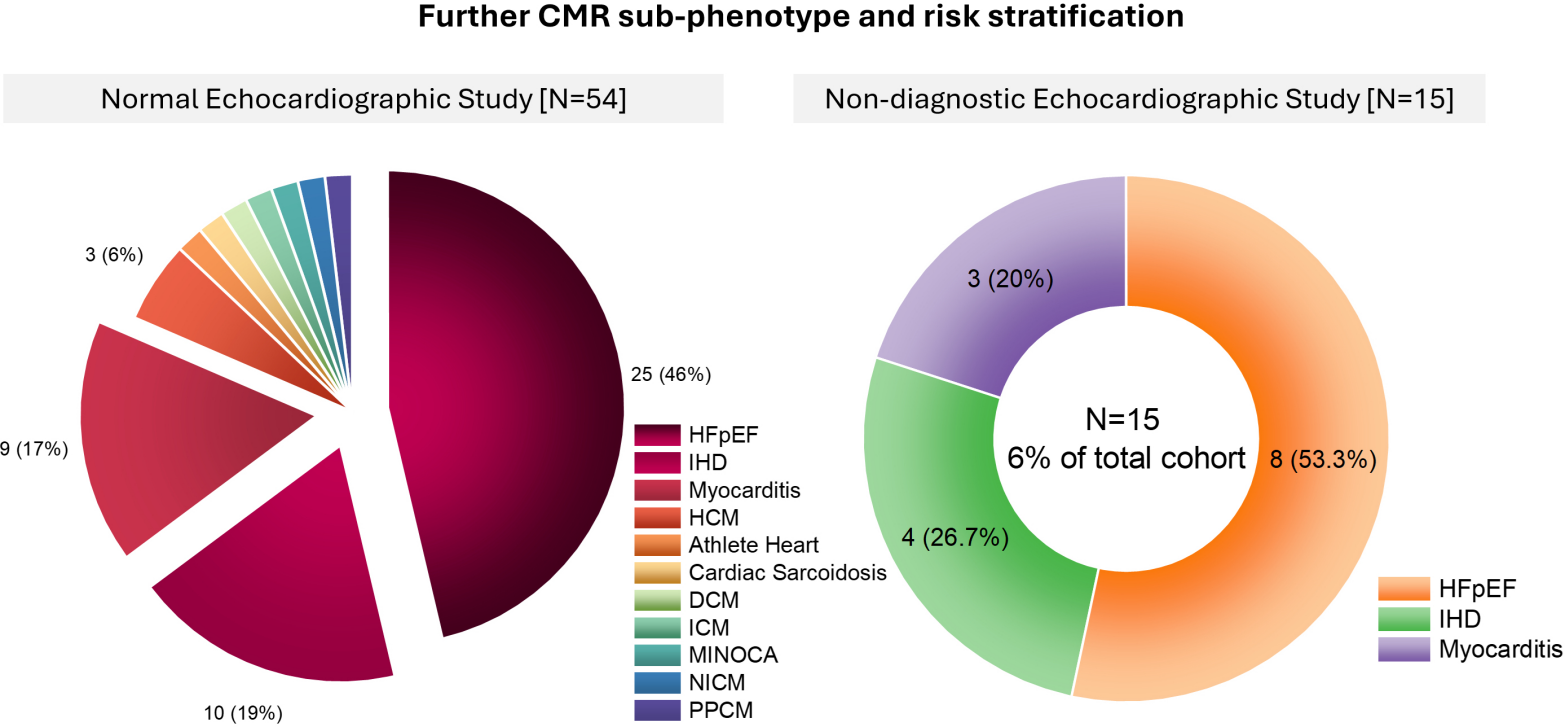
CMR sub-phenotyping in patients with normal or non-diagnostic echocardiography. HFpEF, IHD and myocarditis were the most frequent findings, with additional diagnoses spanning cardiomyopathies and inflammatory conditions. CMR, cardiovascular magnetic resonance; DCM, dilated cardiomyopathy; HCM, hypertrophic cardiomyopathy; HFpEF, heart failure with preserved ejection fraction; ICM, ischaemic cardiomyopathy; IHD, ischaemic heart disease; MINOCA, myocardial infarction with non-obstructive coronary arteries; NICM, non-ischaemic cardiomyopathy; PPCM, peripartum cardiomyopathy.

Among patients with a normal echocardiographic study (n=54), further CMR assessment identified HFpEF as the most prevalent finding, accounting for 46% (n=25) of cases (figure 2, left panel). IHD was the second most common diagnosis, observed in 19% (n=10) of patients, followed by myocarditis in 17% (n=9). Other detected conditions included HCM in 6% (n=3), as well as a range of less frequent diagnoses such as athlete’s heart, cardiac sarcoidosis, dilated cardiomyopathy (DCM), ischaemic cardiomyopathy, myocardial infarction with non-obstructive coronary arteries (MINOCA), non-ischaemic cardiomyopathy (NICM), and peripartum cardiomyopathy (PPCM). These findings highlight the heterogeneous nature of underlying cardiac abnormalities in patients with preserved echocardiographic function despite elevated LVFP.

### Non-diagnostic echocardiography study in patients with raised LVFP by CMR

Among those with a non-diagnostic echocardiographic study (n=15) (figure 2, right panel), CMR provided additional diagnostic clarity, revealing HFpEF in 53.3% (n=8) of cases. IHD was identified in 26.7% (n=4), while myocarditis was detected in 20% (n=3). These results indicate that despite the absence of definitive echocardiographic findings, a substantial proportion of these patients exhibit myocardial pathology detectable on advanced imaging. The predominance of HFpEF within this subgroup further underscores its significant burden in patients with elevated LVFP and non-diagnostic echocardiographic assessments.

### Further sub-phenotyping with CMR

**Figure 3:**
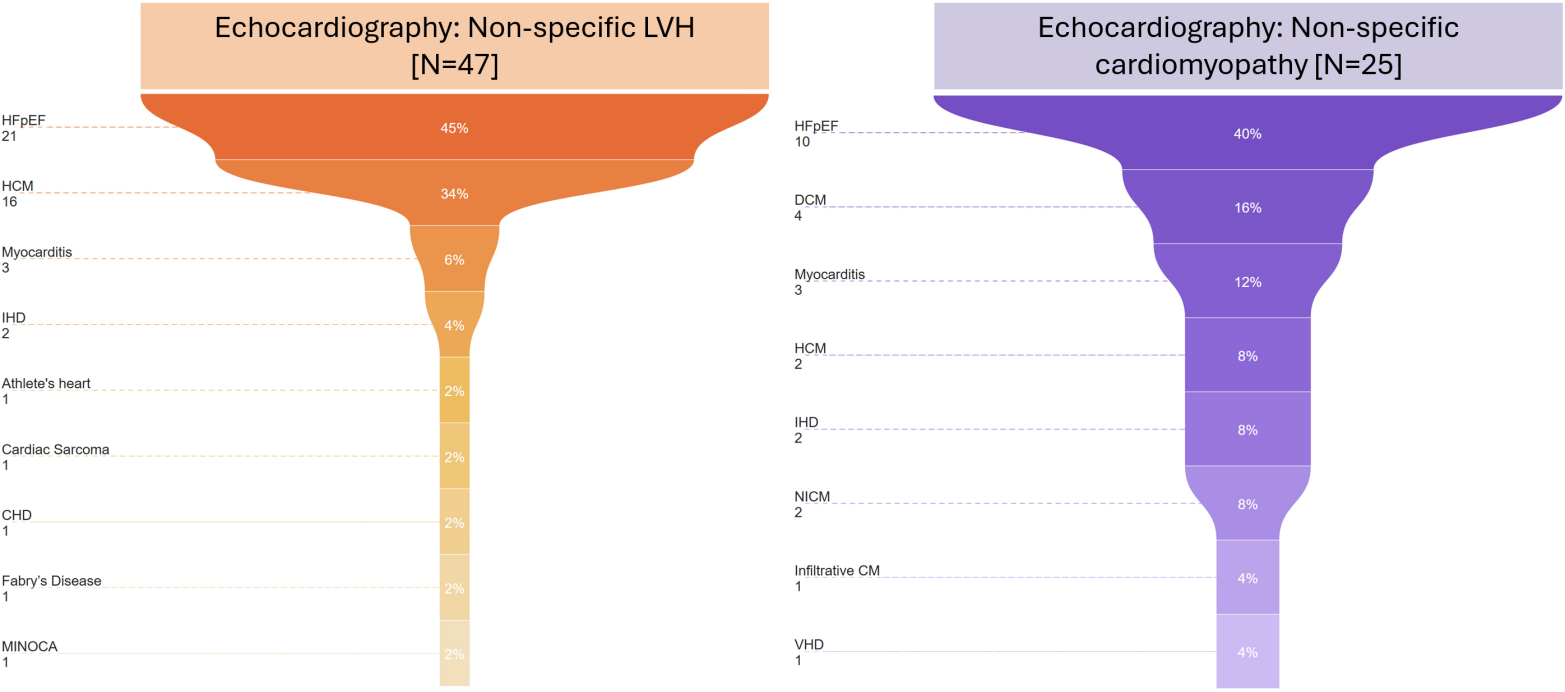
Funnel plot: CMR sub-phenotyping in patients with non-specific LVH and cardiomyopathy. HFpEF was universal, with HCM, myocarditis, IHD and other cardiomyopathies as additional findings. CMR, cardiovascular magnetic resonance; CHD, congenital heart disease; DCM, dilated cardiomyopathy; HFpEF, heart failure with preserved ejection fraction; HCM, hypertrophic cardiomyopathy; IHD, ischaemic heart disease; LVH, left ventricular hypertrophy; MINOCA, myocardial infarction with non-obstructive coronary arteries; NICM, non-ischaemic cardiomyopathy; VHD, valvular heart disease.

Among patients with non-specific LVH on echocardiography (n=47), CMR identified HFpEF in 45% (n=21), highlighting its predominant role in this cohort (figure 3, left panel). HCM was the second most common diagnosis, present in 34% (n=16), followed by myocarditis in 6% (n=3). IHD was observed in 4% (n=2), while athlete’s heart, cardiac sarcoma, congenital heart disease, Fabry’s disease and MINOCA were each found in 2% (n=1), representing a more diverse spectrum of underlying conditions. These findings indicate that a significant proportion of patients with echocardiographic evidence of LVH have underlying myocardial pathology, predominantly HFpEF and HCM, with additional rarer conditions requiring further risk stratification.

Among individuals with non-specific cardiomyopathy on echocardiography (n=25), CMR identified HFpEF in 40% (n=10) (figure 3, right panel). DCM was present in 12% (n=4), and myocarditis was detected in 12% (n=3). HCM and IHD were each observed in 8% (n=2). Additionally, NICM, infiltrative cardiomyopathy and valvular heart disease (VHD) were identified in 4% (n=1) of cases. These data highlight the presence of diverse myocardial pathologies among patients with non-specific cardiomyopathy on echocardiography.

**Figure 4:**
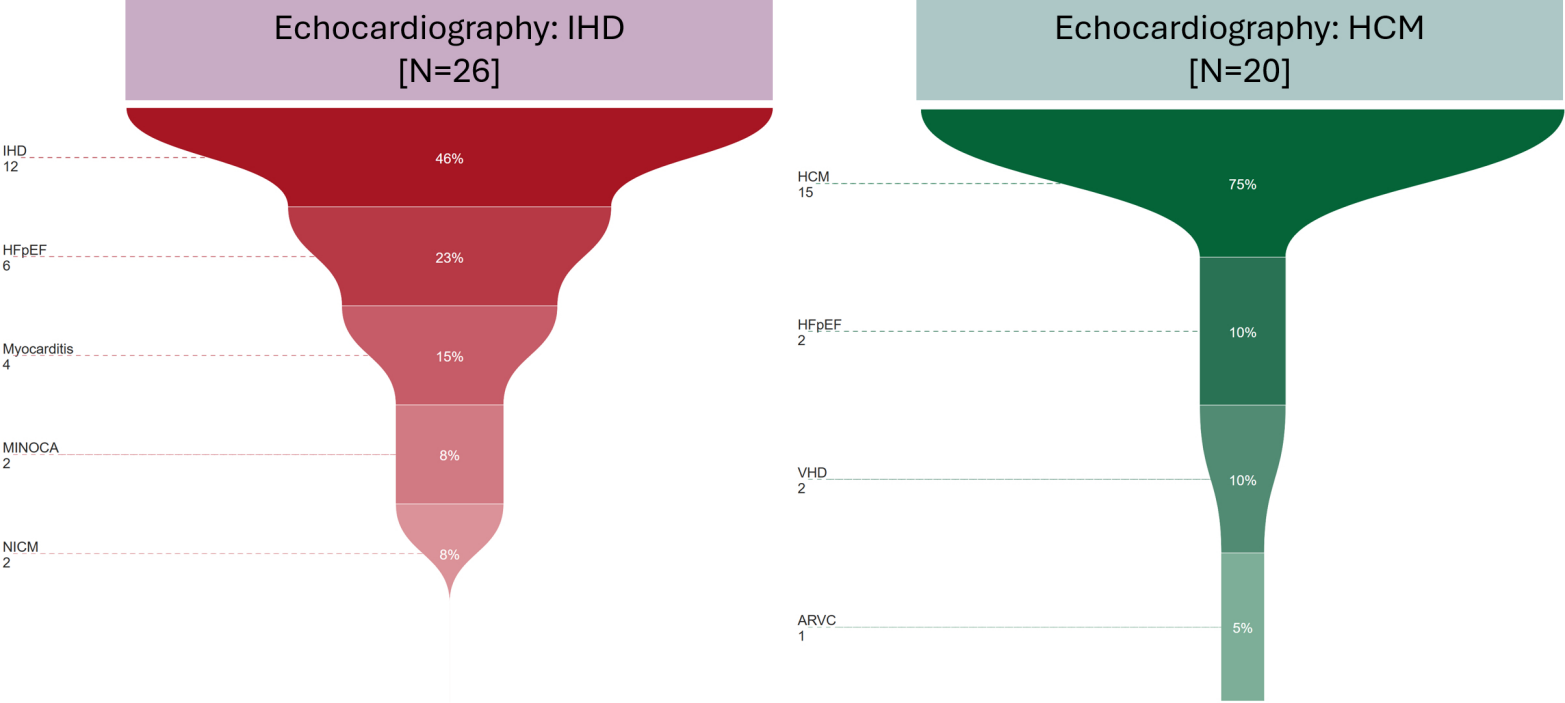
CMR sub-phenotyping in patients with IHD and HCM. CMR confirmed the echocardiographic diagnosis, with additional findings including HFpEF, myocarditis and other cardiomyopathies. ARVC, arrhythmogenic right ventricular cardiomyopathy; CMR, cardiovascular magnetic resonance; HCM, hypertrophic cardiomyopathy; HFpEF, heart failure with preserved ejection fraction; IHD, ischaemic heart disease; MINOCA, myocardial infarction with non-obstructive coronary arteries; NICM, non-ischaemic cardiomyopathy; VHD, valvular heart disease.

Among patients with echocardiographic findings suggestive of IHD (n=26), CMR confirmed the diagnosis in 46% (n=12) of cases (figure 4, left panel). HFpEF was identified in 23% (n=6), while myocarditis was detected in 15% (n=4). MINOCA and NICM were each observed in 8% (n=2) of cases. These findings illustrate the spectrum of myocardial abnormalities that may coexist or even refute the original diagnosis of IHD by echocardiographic examination.

In the cohort with echocardiographic findings consistent with HCM (n=20), CMR confirmed the diagnosis in 75% (n=15) of cases (figure 4, right panel). Additional findings included HFpEF in 10% (n=2) and VHD in 10% (n=2), reflecting the structural and functional heterogeneity within this group. Arrhythmogenic right ventricular cardiomyopathy (ARVC) was identified in 5% (n=1), suggesting overlapping cardiomyopathic features in certain patients. These results highlight the role of CMR in refining diagnostic classifications and detecting concurrent cardiac conditions in patients with suspected HCM.

## Discussion

The most important finding of this study is that CMR provides substantially greater diagnostic yield than TTE, both in refined sub-phenotyping and in aiding definitive diagnosis. CMR consistently identified specific underlying pathologies in patients for whom TTE offered only nonspecific or inconclusive findings, thereby converting broad echocardiographic categories into precise diagnoses. Notably, among patients in whom TTE could only describe unexplained LVH or indeterminate cardiomyopathy, CMR uniformly revealed an underlying HFpEF and further differentiated a spectrum of conditions such as HCM, myocarditis, DCM and infiltrative cardiomyopathies—critical pathologies that were not recognised on echocardiography. Even when TTE yielded a presumptive diagnosis (eg, IHD or HCM), CMR not only confirmed the primary finding but also frequently uncovered additional abnormalities, including concomitant HFpEF, myocardial inflammation or valvular disease, that were missed by TTE. These direct comparisons highlight the intrinsic limitations of echocardiography in fully characterising complex cardiac disease and underscore CMR’s role as a complementary and essential diagnostic test in this setting.

Our findings align with and extend the evidence from other large, registry-based studies on the clinical utility of CMR. For instance, a report from the SCMR Registry by Kwong *et al* demonstrated that CMR had a significant impact on the management of patients with HF.^14^ While their work highlighted downstream changes in therapy and clinical decision-making, our study provides a more focused, upstream analysis of the diagnostic process itself. We specifically quantify the high rate of diagnostic discordance (74%) between TTE and CMR in a high-risk cohort with elevated LVFP and detail the precise aetiological sub-phenotyping that CMR provides. Our work therefore complements the findings from the SCMR Registry by clarifying the fundamental diagnostic refinement that underpins the subsequent changes in patient management.

Our previous study established the first LVFP model using CMR.^8^ In that respective study, a sub-analysis showed that CMR-modelled PCWP was superior to TTE in classifying patients as normal or raised filling pressures (76% vs 25%). Moreover, CMR-modelled PCWP was associated with an increased risk of death (HR: 1.77, p<0.001). In contrast, our current study specifically investigated the sub-phenotyping of HF aetiology in patients already confirmed to have high LVFP (>14 mm Hg) as determined by CMR. Rather than focusing on establishing accuracy, our work aimed to uncover the specific underlying causes of HF. Worryingly, our findings revealed significant limitations in TTE’s diagnostic capabilities. Among our 261 patients with confirmed high LVFP by CMR, TTE identified 21% (n=54) of cases as normal, suggesting no apparent cardiac abnormality despite the presence of elevated LVFP and underlying pathological HF phenotypes. Additionally, in 6% (n=15) of cases, TTE yielded non-diagnostic results. These discrepancies highlight that where TTE may miss or inadequately characterise HF phenotype, CMR assessment should be considered clinically for further assessment.

**Figure 5:**
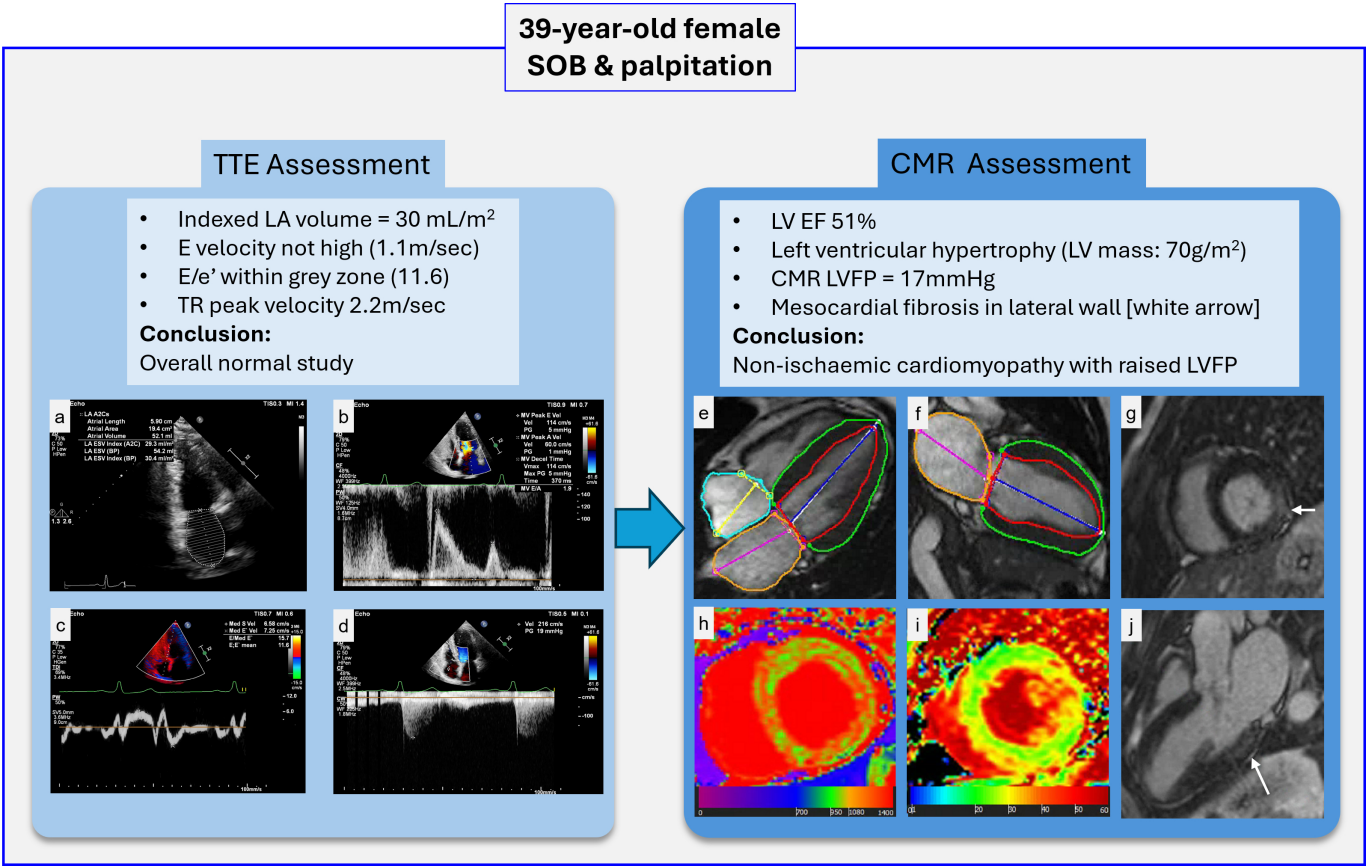
Multimodal cardiac assessment of a 39-year-old female with SOB and palpitations. (**a–d**) TTE shows an indexed LA volume of 30 mL/m², E velocity of 1.1 m/sec, E/e’ of 11.6, and TR peak velocity of 2.2 m/sec, indicating an overall normal study. (**e–g**) CMR cine images demonstrate LVH (LV mass: 70 g/m²) with preserved LVEF (51%). (**h–i**) Native T1 reveals islets of global changes in the myocardium, whereas ECV mapping reveals more focal fibrosis in lateral wall. (**g, j**) LGE imaging highlights mesocardial fibrosis in the lateral wall (white arrows), consistent with NICM. CMR, cardiac magnetic resonance; ECV, extracellular volume; LA, left atrium; LV, left ventricle; LVEF, left ventricular ejection fraction; LVH, left ventricular hypertrophy; LVFP, left ventricular filling pressure; LGE, late gadolinium enhancement; NICM, non-ischaemic cardiomyopathy; SOB, shortness of breath; TTE, transthoracic echocardiography; TR, tricuspid regurgitation.

The contrasting findings between Rahi *et al*’s study,^15^ which TTE high accuracy in estimating LVFP, and the attached study emphasising CMR superior diagnostic sub-phenotyping, underscore the nuanced interplay between modality-specific strengths and clinical context. While Rahi *et al* may demonstrate TTE’s precision in LVFP quantification—potentially due to optimised Doppler parameters, standardised protocols, or a cohort with less heterogeneous pathologies—the attached study reveals CMR’s unmatched ability to unravel complex myocardial pathologies in patients with elevated LVFP, where TTE yielded normal or nonspecific results in 27% of cases. Crucially, diagnostic value transcends singular parameter accuracy; CMR’s capacity to detect HFpEF, myocarditis or infiltrative diseases—conditions with distinct management implications—elevates its clinical utility despite comparable LVFP metrics (figure 5). Ultimately, while TTE remains indispensable for initial haemodynamic assessment, CMR emerges as an irreplaceable adjunct, transforming diagnostic ambiguity into actionable precision—a paradigm where anatomical resolution and tissue interrogation outweigh isolated parameter fidelity.

### Guidelines and clinical context

Our present study aligns robustly with the 2023 European Society of Cardiology (ESC) guidelines for the management of cardiomyopathies, which endorse CMR as Class I (Level of Evidence: B) for diagnosing & phenotyping cardiomyopathies, particularly when echocardiography is inconclusive or when tissue characterisation is critical.^16^ The ESC emphasises CMR’s unparalleled ability to detect myocardial fibrosis (via LGE), inflammation and infiltrative pathologies—key features that guide risk stratification and therapy. This study amplifies these guidelines by demonstrating CMR’s diagnostic superiority in a real-world cohort of patients with elevated LVFP, where 74% of cases showed diagnostic discordance between CMR and TTE. Specifically, CMR identified occult HFpEF, IHD and cardiomyopathies (eg, HCM, myocarditis) in 46%–53% of patients with normal or non-diagnostic TTE, directly addressing the ESC’s call for advanced imaging to resolve diagnostic ambiguity. While the ESC acknowledges TTE’s role in initial haemodynamic assessment, it underscores CMR’s necessity for comprehensive phenotyping—a paradigm this study validates by highlighting CMR’s capacity to uncover pathologies with distinct therapeutic implications (eg, fibrosis-directed therapies in HCM, immunosuppression in myocarditis). The work also reinforces the ESC’s emphasis on multimodality imaging, as CMR’s tissue-level insights complement TTE’s functional data, ensuring holistic patient management. By providing prospective, registry-based evidence of CMR’s incremental value, this study elevates the ESC’s Level B evidence toward broader applicability, advocating for CMR’s integration into routine practice for high-risk cohorts where precision medicine is paramount. Thus, the findings not only mirror but dynamically extend the ESC’s framework, cementing CMR as an indispensable tool in the era of individualised cardiovascular care.

### Limitations

This study has several limitations. The cohort was derived from a single-centre prospective registry, which may introduce referral bias, as only patients undergoing both TTE and CMR were included. While this may skew the cohort toward more diagnostically challenging cases, this approach also reflects a shift in real-world clinical practice that is aligned with current international guidelines. For instance, the 2023 ESC guidelines for cardiomyopathies advocate for the robust use of CMR in this patient population.^16^ Therefore, our cohort is representative of patients undergoing guideline-directed, multi-modality imaging for diagnostic uncertainty. The focus on individuals with elevated CMR-derived LVFP limits generalisability to populations with normal filling pressures or early-stage disease, yet this targeted approach is crucial for evaluating the utility of CMR in high-risk cohorts where accurate phenotyping has the greatest clinical impact. Additionally, non-invasive surrogates were used for LVFP estimation, with CMR-derived PCWP and echocardiographic indices serving as proxies. While invasive haemodynamic validation was not performed, we acknowledge that while echocardiographic indices for LVFP are widely used, the CMR-derived PCWP method is an emerging technique. The study therefore provides valuable insights into their comparative performance in routine diagnostic pathways. Furthermore, we acknowledge that much of the foundational work on this specific CMR-derived model has been conducted by our research group, and broader, multi-centre validation by independent groups is an important next step to confirm its wider generalisability. The lack of simultaneous imaging may have introduced variability due to interim changes in volume status or therapy, but this reflects real-world clinical conditions where imaging is often performed sequentially rather than concurrently.

Another key limitation is the absence of an independent reference standard to adjudicate discrepancies between CMR and TTE. CMR often identified more specific diagnoses when TTE was inconclusive, potentially biasing definitions of diagnostic discordance in favour of CMR. However, this finding highlights CMR’s added diagnostic value in patients with indeterminate TTE results, reinforcing its role as a necessary adjunct in unclear cases. Given the inherent differences in imaging capabilities, some discrepancies may reflect modality-specific detection rather than true clinical uncertainty, yet this underscores CMR’s ability to detect myocardial pathology beyond TTE’s resolution. Additionally, subgroup analyses were based on relatively small patient subsets, limiting statistical power, but the consistency of findings across groups strengthens the robustness of the conclusions.

Another limitation is the absence of systematically collected clinical outcome data for this cohort. While our study robustly quantifies the diagnostic reclassification provided by CMR, we did not analyse subsequent changes in patient management or long-term outcomes such as HF hospitalisations or mortality. Therefore, the direct impact of this improved diagnostic precision on clinical events remains a crucial area for future investigation.

Despite these limitations, the study provides compelling evidence that CMR serves as a complementary and essential diagnostic tool, refining diagnostic accuracy and sub-phenotyping beyond TTE alone.

## Conclusions

In conclusion, this study demonstrates that CMR enhances diagnostic precision and sub-phenotyping in patients with raised LVFP, particularly when TTE is normal or non-diagnostic. CMR consistently identifies critical pathologies, including myocarditis, IHD and cardiomyopathies, that echocardiography alone cannot detect. These findings underscore the essential role of CMR in refining diagnosis and guiding clinical management.

## Data Availability

Data are available upon reasonable request.
