# Supplementary material for: Does CMR improve aetiological sub-phenotyping beyond echocardiography in patients with elevated LV filling pressure? A prospective registry study (PREFER-CMR)
Source: BMJ Open. 2026 Jan 14;16(1):e102836. doi: 10.1136/bmjopen-2025-102836 (PMC12815195; doi:10.1136/bmjopen-2025-102836)
Supplement: Supplementary file 1 [file bmjopen-2025-102836.reviewer_comments.pdf]

PEER REVIEW HISTORY

BMJ Open publishes all reviews undertaken for accepted manuscripts. Reviewers are asked to complete a checklist review form and are provided with free text boxes to elaborate on their assessment. These free text comments are reproduced below.

ARTICLE DETAILS

Title (Provisional)

Does CMR improve aetiological sub-phenotyping beyond Echocardiography in patients with elevated LV filling pressure? A prospective registry study (PREFER-CMR)

Authors

Bana, Aradhai; Li, Rui; Mehmood, Zia; Rogers, Craig; Grafton-Clarke, Ciaran; Bali, Tiya; Hall, David; Jamil, Mustapha; Ramachenderam, Liandra; Dudhiya, Uwais; Spohr, Hilmar; Underwood, Victoria; Girling, Rebekah; Kasmai, Bahman; Nair, Sunil; Matthews, Gareth; Garg, Pankaj

---

VERSION 1 - REVIEW

**Reviewer**	**1**
**Name**	**Backhaus, Sören J.**
**Affiliation**	**Kerckhoff Clinic**
**Date**	**19-Jun-2025**
**COI**	**None**

In their manuscript, the Bana et al. investigate the ability of CMR to identify heart failure aetiologies beyond echocardiography. The authors present a comprehensively characterised registry population.

With CMR being the reference standard for cardiac morphological and functional characterisation, it is very intriguing to see 74% of 261 patients emerge with non-similar diagnosis following echocardiographic and CMR assessments. The strength of the present work is the detailed presentation of improved identification of specific HF subtypes by CMR as opposed to TTE including amongst others ischaemic heart disease and cardiomyopathies.

The major limitation of the manuscript lies within characterisation of elevated filling pressures and diastolic dysfunction.

- The most major limitation of the present manuscript is the absence of invasive pressure measurements – especially considering the authors primarily address patients with LVFP of >14mmHg. Right heart catheterisation-derived PCWP represents the reference standard

amongst all current guideline recommendations for the identification of elevated filling pressures.

- LVFP calculation is based on CMR-derived measurements only. All references given to demonstrate their value come from their own working group. Whilst, this is very briefly addressed in the limitation section, stating CMR derived PCWP is widely used is kind of an overstatement.

- Echocardiography represents the clinical go to imaging standard, with E/e' representing the basis - although consistently challenged - for diastolic function evaluation. All current guideline recommendations for the echocardiographic evaluation of HFpEF include E/e' as a cornerstone. The method section reads E/e' has been assessed, though I can't seem to find E/e' neither in the result section nor Table 2.

Please could the authors elaborate on what a non-diagnostic echo specifically refers to?

I am aware that in the setting of this retrospective analysis of registry data to test the present hypothesis the lack of RHC-derived PCWP cannot be addressed. However, the wording on the soundness and diagnostic accuracy of CMR-derived PCWP is to strong. With the classification of elevated LVFP based upon this methodology alone, the derived conclusion and its wording may not be adequate.

**Reviewer**	**2**
**Name**	**Roifman, Idan**
**Affiliation**	**Sunnybrook Health Sciences Centre, Medicine**
**Date**	**24-Sep-2025**
**COI**	**None**

This prospective study compared echocardiography (TTE) and cardiac MRI (CMR) in 261 patients with CMR derived elevated left ventricular filling pressure. CMR identified significant pathologies missed by TTE, including HFpEF, ischemic heart disease, and myocarditis. Findings show CMR improves diagnostic precision, refining sub-phenotyping and informing targeted management strategies. The following should be considered by the authors:

1. Overall, the paper adds to the growing body of work on the added utility of CMR imaging. The authors should include other large resgistry-based published works in their discussion that reported on the added value in CMR with regards to changing diagnoses. For example, the following paper should be discussed and the authors should mention what their study contributes above and beyond it:

Utilization and impact of cardiovascular magnetic resonance on patient management in heart failure: insights from the SCMR Registry. J Cardiovasc Magn Reson 24, 65 (2022). https://doi.org/10.1186/s12968-022-00890-0

2. Are there any clinical correlates available? In other words, do the authors know if any of the patients were in clinical heart failure which would correspond with the high filling pressures/HFPEF diagnoses?

3. Are there data available on patient outcomes? It would be interesting to determine if a change in diagnosis as a result of CMR was associated with a change in clinical outcomes.

4. The study was conducted at one UK institution, limiting generalisability to broader, more diverse populations.

5. Only patients referred for both TTE and CMR were included in the study, potentially skewing the cohort toward more complex cases.

6. LVFP was estimated using non-invasive methods with no gold standard utilized (invasive catheterization). This is especially problematic for CMR-derived LVFP calculation. This is rarely used clinically and has many potential errors and pitfalls in its calculation.

7. How many patients had TTE and CMR performed on the same day? Changes in hemodynamics are common and are responsive to medications. A systematic lag between TTE and CMR could impact the results.

VERSION 1 - AUTHOR RESPONSE

Reviewer 1

1. The most major limitation of the present manuscript is the absence of invasive pressure measurements – especially considering the authors primarily address patients with LVFP of >14mmHg. Right heart catheterisation-derived PCWP represents the reference standard amongst all current guideline recommendations for the identification of elevated filling pressures.

Reply: We thank the reviewer for raising this important point. We agree entirely that RHC is the reference standard for the direct measurement of PCWP.
The CMR-derived model used in this study to identify the cohort with elevated LVFP was developed and validated against invasive RHC data in our group's prior work (Garg et al., Eur Heart J, 2022; DOI: 10.1093/eurheartj/ehac133). The primary objective of the current study was therefore not to re-validate this model, but to apply it to a prospective, real-world registry. Our specific aim was to investigate the incremental diagnostic value of CMR for aetiological sub-phenotyping in patients identified by this non-invasive metric, and to compare these findings directly against TTE.
Given that the focus of this paper was on the diagnostic discordance between two imaging modalities within a pre-defined high-risk group, we believe the absence of concurrent invasive data, while a limitation, does not invalidate our primary findings on the comparative diagnostic yield of CMR versus TTE.

2. LVFP calculation is based on CMR-derived measurements only. All references given to demonstrate their value come from their own working group. Whilst, this is very briefly addressed in the limitation section, stating CMR derived PCWP is widely used is kind of an overstatement.

Reply: We thank the reviewer for these observations. We acknowledge that much of the foundational work on this specific CMR-derived LVFP model has been conducted by our research group as part of a focused programme of work in this area. We agree that broader, multi-centre validation by independent groups is an important next step to ensure wider generalisability.
Furthermore, we completely agree with the reviewer's assessment that describing the CMR-derived PCWP method as 'widely used' is an overstatement at this stage. We have taken this feedback on board and have revised the manuscript to more accurately describe it as an 'emerging' technique, reflecting its current state of adoption in clinical and research settings.

3. Echocardiography represents the clinical go to imaging standard, with E/e' representing the basis - although consistently challenged - for diastolic function evaluation. All current guideline recommendations for the echocardiographic evaluation of HFpEF include E/e' as a cornerstone. The method section reads E/e' has been assessed, though I can't seem to find E/e' neither in the result section nor Table 2.

Reply: We thank the reviewer for their diligence in spotting this error and for giving us the opportunity to correct it. The reviewer was correct that the E/e' ratio was not explicitly labeled in our results. This was due to an unfortunate labeling error on our part. The data for the E/e' ratio was indeed present in Table 2 but had been incorrectly labeled as 'Early Diastolic Mitral Annular Velocity'.
We have now corrected this in the revised manuscript. Table 2 has been updated to correctly label this row as 'Mitral inflow E velocity to mitral annular early diastolic velocity ratio (E/e′)'. We have also amended the corresponding sentence in the main results text to accurately describe this finding. We apologise for this oversight and appreciate the reviewer's help in improving the accuracy of our manuscript.

4. Please could the authors elaborate on what a non-diagnostic echo specifically refers to?

Reply: Thank you for this request for clarification. We agree a precise definition is important.
In our clinical practice, echocardiographic image quality is formally graded by the reporting sonographer as part of a standardised protocol. In this framework, a 'non-diagnostic' study is specifically defined as a scan that is deemed by the sonographer to have 'no diagnostic utility'. This is the most severe classification and is distinct from a study of 'limited' quality, which may provide some, but not all, of the required diagnostic information.

5. I am aware that in the setting of this retrospective analysis of registry data to test the present hypothesis the lack of RHC-derived PCWP cannot be addressed. However, the wording on the soundness and diagnostic accuracy of CMR-derived PCWP is to strong. With the classification of elevated LVFP based upon this methodology alone, the derived conclusion and its wording may not be adequate.

Reply: We thank the reviewer for summarising their concerns. As detailed in our earlier response, we have taken this feedback seriously. We have carefully revised the

manuscript to soften the wording regarding the CMR-derived PCWP method, now describing it more accurately as an 'emerging' technique rather than 'widely used'. We believe this revision appropriately moderates the language and addresses the reviewer's valid concerns about the strength of our claims.

Reviewer 2

1. Overall, the paper adds to the growing body of work on the added utility of CMR imaging. The authors should include other large resgistry-based published works in their discussion that reported on the added value in CMR with regards to changing diagnoses. For example, the following paper should be discussed and the authors should mention what their study contributes above and beyond it:
Utilization and impact of cardiovascular magnetic resonance on patient management in heart failure: insights from the SCMR Registry. J Cardiovasc Magn Reson 24, 65 (2022). https://doi.org/10.1186/s12968-022-00890-0
Reply: We thank the reviewer for this constructive suggestion and for providing this highly relevant reference. We agree that discussing our findings in the context of other large registry studies will strengthen our Discussion.
We have now added a paragraph to the Discussion section that references the suggested paper from the SCMR Registry. In this new text, we highlight that while the SCMR Registry study demonstrated the broad impact of CMR on patient management in heart failure, our study provides a more focused analysis on the specific diagnostic discordances and refined aetiological sub-phenotyping provided by CMR over TTE in a high-risk cohort with elevated LVFP. We believe our findings therefore complement their work by detailing the crucial diagnostic step that precedes and informs the changes in management they reported.

2. Are there any clinical correlates available? In other words, do the authors know if any of the patients were in clinical heart failure which would correspond with the high filling pressures/HFPEF diagnoses?
3. Are there data available on patient outcomes? It would be interesting to determine if a change in diagnosis as a result of CMR was associated with a change in clinical outcomes.
Reply: The reviewer raises an excellent and important point regarding the correlation of our imaging findings with clinical heart failure status and subsequent patient outcomes. We agree that this is a critical area for investigation.
The scope of the present study was focused on quantifying the diagnostic yield of CMR and the discordance with TTE. For this specific analysis, we did not systematically collect data on clinical heart failure signs and symptoms or follow-up data on clinical events such as hospitalisations or mortality.
We have now explicitly acknowledged this as a limitation in the manuscript. We state that the absence of clinical outcome data prevents us from determining whether the diagnostic refinement provided by CMR led to changes in management or improved patient outcomes, and we highlight that this is a key objective for future research.

4. The study was conducted at one UK institution, limiting generalisability to broader, more diverse populations.
5. Only patients referred for both TTE and CMR were included in the study, potentially skewing the cohort toward more complex cases.

Reply: We thank the reviewer for raising these valid methodological points. We acknowledge in the manuscript that the single-centre design and the inclusion of only patients who underwent both imaging modalities are limitations.
However, we would like to provide important context for this methodology. Our institution is a tertiary referral centre, and the increasing use of CMR is a direct reflection of a shift in clinical practice that is aligned with major international guidelines. Specifically, the 2023 ESC guidelines for the management of cardiomyopathies now strongly advocate for the robust use of CMR in patients with suspected cardiomyopathy.
Therefore, while our cohort may include more complex cases, it is also increasingly representative of the patient population that is being investigated according to modern, guideline-directed care. Our findings demonstrate the diagnostic utility of CMR in the very population for whom these guidelines now recommend its use.

6. LVFP was estimated using non-invasive methods with no gold standard utilized (invasive catheterization). This is especially problematic for CMR-derived LVFP calculation. This is rarely used clinically and has many potential errors and pitfalls in its calculation.
Reply: We thank the reviewer for highlighting this important methodological point. We agree that our study did not utilise an invasive gold standard for LVFP, and we have clearly stated this as a limitation in the manuscript.
However, the primary purpose of this paper was not to validate the CMR-derived LVFP metric itself. Instead, our research question focused on the diagnostic change and refined sub-phenotyping that occurs when patients with non-invasively estimated high LVFP undergo CMR after an initial TTE. The study's core finding is the significant diagnostic discordance between these two imaging modalities within this specific, high-risk cohort.
Therefore, while the absence of a gold standard for the initial cohort identification is a valid limitation, it does not invalidate our primary findings, which pertain to the comparative diagnostic value of CMR versus TTE in this setting.

7. How many patients had TTE and CMR performed on the same day? Changes in hemodynamics are common and are responsive to medications. A systematic lag between TTE and CMR could impact the results.
Reply: The reviewer raises a very important practical point regarding the timing of the imaging studies. We agree that haemodynamic parameters can fluctuate.
However, the primary objective of our study was to compare the final, overall aetiological diagnosis derived from a comprehensive TTE study versus a comprehensive CMR study, rather than performing a direct quantitative comparison of specific haemodynamic metrics between the two modalities. A fundamental diagnosis, such as the presence of hypertrophic cardiomyopathy or myocardial scar, is a stable finding and is far less likely to change over the short interval between scans. Therefore, we believe that non-simultaneous scanning does not invalidate our findings on diagnostic reclassification.
Nevertheless, we can reassure the reviewer that, in line with standard clinical practice, the CMR was typically performed in close temporal proximity to the TTE as part of the same diagnostic workup. We would also like to respectfully point out that we proactively acknowledged this in the Limitations section, where we state: 'The lack of simultaneous imaging may have introduced variability due to interim changes in

volume status or therapy, but this reflects real-world clinical conditions where imaging is often performed sequentially rather than concurrently'.
